# Evaluation of postoperative pain in patients submitted to periodontal surgeries

**DOI:** 10.1080/07853890.2021.1897343

**Published:** 2021-09-28

**Authors:** José Maria Cardoso, Miguel Ribeiro, Ricardo Alves

**Affiliations:** a Perodontology Department, Instituto Universitário Egas Moniz (IUEM), Egas Moniz Cooperativa de Ensino Superior, Caparica, Portugal; b Department of Dental Medicine, Instituto Universitário Egas Moniz (IUEM), Egas Moniz Cooperativa de Ensino Superior, Caparica, Portugal

## Abstract

**Introduction:**

Different periodontal surgeries are used to treat a variety of periodontal conditions in favour of a healthy and aesthetic periodontium [1]. However, the fear of surgical treatment is common, most often depriving patients of undergoing complete dental treatments. Patients' questions and concerns are often pain-related [2]. Adequate understanding of the intensity and variables that affect pain is essential because it can produce emotional responses that may influence treatment adherence [3]. The objectives of this study are evaluation of pain after periodontal surgery and its relationship with variables related to the patient, surgery and postoperative care.

**Materials and methods:**

This study was approved by the Egas Moniz Ethics Committee and by the Direction of the Egas Moniz University Clinic (CUEM). All patients referred in the sample signed an informed consent form. Questionnaires were applied to 63 patients submitted to periodontal surgeries at the Post-graduation Course of Periodontology at the CUEM. Data was collected through the completion of two questionnaires. The first questionnaire was composed of two parts and was applied in the presence of the patient. The part A was done on the day of surgery for the purpose of collecting data from the patient’s clinical history and related with the surgery; and the part B on the day of suture removal about post-operative care. The second was delivered on the day of surgery with the Visual Analogue Scale. The patient was asked to fill it on the day of surgery, on the next two days after surgery and also on the day of suture removal, returning it on that day.

**Results:**

It was found that the highest pain levels were experienced by the patients on the day of surgery, with a median value of 6.9. The degree of postoperative pain is not related with gender, type of periodontal disease, type of periodontal surgery, technique performed, teeth involved, duration of surgery, antibiotic intake, use of chlorhexidine gel and mouthwash, absence of mechanical plaque control and absence of physical exercise. On the other hand, the degree of postoperative pain is dependent on factors such as age (with older patients experiencing less pain), smoking habits and smoking cessation (non-smoking patients or those who stopped the habit in the postoperative period showed less postoperative pain). The use of nonsteroidal anti-inflammatory drugs and its duration influence the postoperative pain.

**Discussion and conclusions:**

Periodontal treatment often includes several surgical procedures, so a bad postoperative period may prevent the patient from continuing treatment, which may in some cases jeopardise the maintenance of teeth. Smoking habits have been shown to be the most prominent variable in postoperative pain. It is therefore extremely important to encourage patients to stop smoking or when it is not possible to interrupt during the postoperative period.

## References

[1] American Academy of Periodontology. Comprehensive periodontal therapy: a statement by the American Academy of Periodontology. J Periodontol. 2011;82(7):943–949. 21721992 10.1902/jop.2011.117001

[2] Mei CC, Lee FY, Yeh HC. Assessment of pain perception following periodontal and implant surgeries. J Clin Periodontol. 2016;43(12):1151–1159. 27554366 10.1111/jcpe.12618

[3] Beaudette JR, Fritz PC, Sullivan PJ, et al. Investigation of factors that influence pain experienced and the use of pain medication following periodontal surgery. J Clin Periodontol. 2018;45(5):578–585. 29500837 10.1111/jcpe.12885 PMC5969096

